# Relationship between Glaucoma Drainage Device Size and Intraocular Pressure Control: Does Size Matter?

**DOI:** 10.5005/jp-journals-10008-1212

**Published:** 2017-01-18

**Authors:** Cooper D Rodgers, Alissa M Meyer, Mark B Sherwood

**Affiliations:** 1 Research Assistant, Department of Ophthalmology, University of Florida Gainesville, Florida, USA; 2 Research Assistant, Department of Ophthalmology, University of Florida Gainesville, Florida, USA; 3 Professor, Department of Ophthalmology, University of Florida Gainesville, Florida, USA

**Keywords:** Baerveldt, Glaucoma, Glaucoma drainage device, Intraocular pressure, Molteno, Retrospective study, Visual acuity.

## Abstract

There is ambiguity in the literature regarding whether a larger glaucoma drainage device (GDD) achieves a lower long-term intraocular pressure (IOP). There is some evidence on both sides, but overall there seems to be an optimal surface area of approximately 200-250 mm^2^ beyond which there may be little advantage to increasing the plate size for most patients.

**How to cite this article:**

Rodgers CD, Meyer AM, Sherwood MB. Relationship between Glaucoma Drainage Device Size and Intraocular Pressure Control: Does Size Matter? J Curr Glaucoma Pract 2017;11(1):1-2.

## INTRODUCTION

Over the years, there has been a lack of clarity in the literature regarding the relationship between glaucoma drainage device (GDD) size and intraocular pressure (IOP) control. An early randomized prospective study by Heuer et al^1^ suggested that the double plate Molteno (270 mm^2^) provided better IOP control than the single plate (135 mm^2^) at 1 to 2 years postoperative. A later paper by Britt et al^2^ demonstrated that a larger implant is not necessarily better than a smaller one; the Baerveldt 350 mm^2^ was superior to the Baerveldt 500 mm^2^ in regulating IOP. Does this mean that there is an optimal implant size? In a number of prospective randomized controlled studies, the Baerveldt 350 mm^2^ has been the default implant. The tube *vs* trabeculectomy (TVT) and the primary tube *vs* trabeculectomy (PTVT) study groups^3^ utilized this implant in a comparison of tube shunt surgery and trabeculectomy with mitomycin C (MMC).

In the Ahmed-Baerveldt comparison (ABC) and Ahmed *vs* Baerveldt (AVB) studies,^4,5^ groups compared the silicone Baerveldt 350 mm^2^ with the silicone Ahmed FP7 glaucoma valve (184 mm^2^). These two studies showed that the Baerveldt 350 mm^2^ implant offered superior IOP control, but it is still unclear if this is because of the difference in size of the implants or whether it is related to the fact that the aqueous is delayed in getting to the episcleral plate area with the Baerveldt for about 6 weeks postoperative compared with immediate plate delivery with the Ahmed.

There has been some evidence of increased postoperative diplopia with large implants,^6^ and the Baerveldt was modified in the late 1990s by making fenestrations in the plate in order to lower the height of the bleb. This was achieved by allowing fibrous plugs to grow through these fenestrations, connecting the capsule below and above the plate. The wings of the Baerveldt 350 mm^2^ implant are generally placed underneath two of the recti muscles, which often requires mechanical hooking and manipulation of the muscles at surgery. In the TVT study group, the incidence of diplopia in the 350 mm^2^ Baerveldt group was 5% (5 patients) at 1 year.

There have been several small retrospective studies that have compared the Baerveldt 250 mm^2^ and Baerveldt 350 mm^2^ implants. A 2003 study by Seah et al^7^ reported that there were no statistically significant differences in success rate, complication rate, final IOP, visual acuity (VA), and number of medications between the 350 and 350 mm^2^ implant groups in Asian eyes at a mean follow-up of 33.4 months. Similarly, in 2015, Allan et al^8^ found no significant differences between the two implant sizes at a mean follow-up of 40 months.

In our study,^9^ we have compared the larger Baerveldt 350 mm^2^ implant to the Baerveldt 250 mm^2^ and the Molteno 3 (245 and 230 mm^2^) implants, all of which are placed in a single quadrant. Like previous studies, our study found no significant difference in mean IOP, medication use, or VA change between the larger 350 mm^2^ and the medium 230 to 250 mm^2^ implants.

In conclusion, there does not seem to be good evidence that suggests there are any advantages in using the 350 mm^2^ Baerveldt over smaller 230 to 250 mm^2^ GDDs and, at up to 3 years postoperative, the IOP, VA, and medication use appear similar.
